# Supplementary material for: Major infections following pediatric cardiac surgery pre- and post-CLABSI bundle implementation
Source: PeerJ. 2022 Oct 28;10:e14279. doi: 10.7717/peerj.14279 (PMC9620976; doi:10.7717/peerj.14279)
Supplement: Table S1 [file peerj-10-14279-s001.docx]

**Supplementary data: Major infections following pediatric cardiac surgery pre- and post-CLABSI bundle implementation**

**S1 Table.** Proportion of patients with/without major postoperative infection (n=548)

| **Variables** | **Presence of postoperative major infection (n=126)** | **No major infection**  **(n=422)** |
| --- | --- | --- |
| Male gender | 72 (57.1%) | 221 (52.3%) |
| Age at surgery < 6 months | 59 (46.8%) | 63 (14.9%) |
| Weight < 5 kg | 56 (44.4%) | 67 (15.8%) |
| Presence of genetic syndrome | 17 (13.4%) | 52 (12.3%) |
| Single ventricle | 26 (20.6%) | 52 (12.3%) |
| Asplenia or polysplenia | 14 (11.1%) | 28 (6.6%) |
| History of major infection within 3 months prior to operation | 35 (27.7%) | 44 (10.4%) |
| Preoperative functional class III-IV | 73 (57.9%) | 141 (33.4%) |
| Preoperative usage of ventilator | 18 (14.2%) | 14 (3.3%) |
| Preoperative usage of central line | 31 (24.6%) | 25 (5.9%) |
| Procedure Aristotle Basic Complexity score > 9 | 21 (16.6%) | 38 (9.0%) |
| Type of surgery : emergency or urgency%) | 11 (8.7%) | 11 (2.6%) |
| Operative time > 240 min | 38 (30.1%) | 83 (19.6%) |
| CPB time > 90 min | 69 (54.7%) | 195 (46.2%) |
| Delayed sternal closure | 17 (13.4%) | 15 (3.5%) |
| Ventilator usage > 2 days | 92 (73.0%) | 110 (26.0%) |
| Central line usage > 4 days | 116 (92.1%) | 264 (62.5%) |
| Pre CLABSI bundle period | 74 (58.7%) | 212 (50.2%) |

Data represented by n (%)

min=minutes, CPB=cardiopulmonary bypass; CLABSI=central-line associate bloodstream infection
