# Supplementary material for: Major infections following pediatric cardiac surgery pre- and post-CLABSI bundle implementation
Source: PeerJ. 2022 Oct 28;10:e14279. doi: 10.7717/peerj.14279 (PMC9620976; doi:10.7717/peerj.14279)
Supplement: Table S2 [file peerj-10-14279-s002.docx]

**Supplementary data: Major infections following pediatric cardiac surgery pre- and post-CLABSI bundle implementation**

**S2 table** Risk factors of mortality in postoperative pediatric cardiac surgery (n=548)

| **Variables** | **Crude OR**  **(95% CI)** | **p-value** | **Adjusted OR**  **(95% CI)** | **P-value** |
| --- | --- | --- | --- | --- |
| Male gender | 1.334  (0.638, 2.790) | 0.442 |  |  |
| Age at surgery < 6 months | 6.944  (3.215, 15.151) | <0.001* | 2.462  (0.754, 8.035) | 0.135 |
| Weight < 5 kg | 8.064  (3.649, 17.543) | <0.001* |  |  |
| Presence of genetic syndrome | 1.314  (0.388, 4.453) | 0.661 |  |  |
| Single ventricle | 3.897  (1.776, 8.550) | <0.001* | 6.633  (1.758, 25.018) | 0.005* |
| Asplenia or polysplenia | 3.347  (1.386, 8.712) | 0.009* | 0.548  (0.114, 2.638) | 0.453 |
| History of major infection within 3 months prior to operation | 5.237  (2.432, 11.276) | <0.001* | 1.578  (0.511, 4.879) | 0.428 |
| Preoperative functional class III-IV | 3.978  (1.764, 8.753) | <0.001* | 1.519  (0.510, 4.526) | 0.452 |
| Preoperative usage of ventilator | 11.277  (4.718, 26.933) | <0.001* | 1.648  (0.459, 5.914) | 0.444 |
| Preoperative usage of central line | 8.447  (3.846, 18.581) | <0.001* | 1.716  (0.479, 6.146) | 0.407 |
| Procedure Aristotle Basic Complexity score > 9 | 4.786  (2.120, 10.862) | <0.001* | 1.526  (0.407, 5.721) | 0.531 |
| Emergency or urgency procedure | 10.260  (3.793, 27.458) | <0.001* | 5.350  (1.245, 22.988) | 0.024* |
| Operative time > 240 min | 4.495  (2.126, 9.503) | <0.001* | 3.790  (1.196, 12.006) | 0.024* |
| CPB time > 90 min | 2.246  (1.031, 4.892) | 0.037* | 1.326  (0.390, 4.508) | 0.651 |
| Delayed sternal closure | 9.224  (3.804, 22.363) | <0.001* | 0.821  (0.219, 3.085) | 0.771 |
| Ventilator usage > 2 days | 5.164  (2.254, 11.832) | <0.001* | 1.925  (0.502, 7.388) | 0.341 |
| Central line usage > 4 days | 2.296  (0.863, 6.124) | 0.087 | 0.452  (0.103, 1.981) | 0.292 |
| Pre CLABSI bundle period | 0.953  (0.456, 1.992) | 0.897 |  |  |
| Major infection | 3.172  (1.503, 6.699) | 0.002* | 1.315  (0.499, 3.465) | 0.579 |

Multivariate analysis by logistic regression

* Statistical significance at p-value < 0.05

OR=Odd ratio; min=minutes; CPB=cardiopulmonary bypass; CLABSI= central-line associated blood stream infection
