# Supplementary material for: Major infections following pediatric cardiac surgery pre- and post-CLABSI bundle implementation
Source: PeerJ. 2022 Oct 28;10:e14279. doi: 10.7717/peerj.14279 (PMC9620976; doi:10.7717/peerj.14279)
Supplement: Supplemental Information 3 [file peerj-10-14279-s003.pdf]

Thesis proposal (English version)

Major infections following pediatric cardiac surgery in Siriraj Hospital; revisited post CLABSI bundle

Background and rationale

Fever above 38°C (100.4°F) is common in the first few days after major surgery. Most early postoperative fever is caused by the inflammatory stimulus of surgery and resolves spontaneously. However, postoperative fever can be a manifestation of a serious complication. Differential diagnosis of postoperative fever includes infectious and noninfectious etiologies. Surgical site infection (SSI) and hospital-related infections including nosocomial pneumonia, urinary tract infection, have been known as common infectious causes. On the other hand, noninfectious etiology such as atelectasis, drug fever and deep vein thrombosis, have been recognized as causes of postoperative fever. In evaluating postoperative patients who have fever, a broad differential, not to assume that fever is only due to infection, is crucial. Notably, fever as a manifestation of infection may be reduced or absent in immunocompromised patients, including those receiving glucocorticoids, cancer chemotherapy, post-transplant immunosuppression, and also in some patients who are elderly or have chronic renal failure.

The timing of fever after surgery is one of the most significant factors to consider in generating a prioritized differential diagnosis of postoperative fever. The timing of postoperative fever can be usefully described as:

- Immediate: onset in the operating suite or within hours after surgery
- Acute: onset within the first week after surgery
- Subacute: onset from one to four weeks following surgery
- Delayed: Onset more than one month after surgery

Although the list of causes of postoperative fever is extensive, the initial focus for most patients should be on a limited number of the more common infectious and noninfectious causes. Common infectious causes are surgical site infection, pneumonia, urinary tract infection, and septicemia. Common noninfectious causes are Drug fever, Deep vein thrombosis, Seroma, and surgical site reaction.(1)

Siriraj Hospital is a tertiary university hospital and the main referral hospital for congenital cardiac surgery in Thailand. Case volumes of congenital cardiac surgery are 300 - 400 cases/year, with up to 70% for patients in the pediatric age group. Databases of patients 0 to 15 years old, who underwent cardiac surgery in Siriraj Hospital between January 1 and December 31, 2005 were retrospectively reviewed. The main outcomes of interest were postoperative fever and its etiologies. Potential predictors were analyzed by comparing patients who developed or did not have infections. The results are Two hundred thirty patients, 43% (n = 99) developed postoperative fever. Major infections occurred in 13.5% (n = 31), and postpericardiotomy syndrome (PPS) was seen in 8.7% (n = 20) of the patients. The infection rate was 16.9/100 procedures, including pneumonia (29 episodes) and blood stream infection (6 episodes). Risk factors were infancy, prolonged ventilator support > 2 days, hospital length of stay (LOS) > 14 days, intensive care unit (ICU) LOS > 3 days, re-open procedure, and extubation failure rate. Conversely, cyanosis and high complexity operations were not associated. Positive erythrocyte sedimentation rate was related to infections or to PPS (the area under the ROC = 0.72).(2)

Based on the Society of Thoracic Surgeons Congenital Heart Surgery Database, patients less than 18 years of age at operation from January 1, 2002, to December 31, 2006 were retrospectively reviewed. There were 30,078 children from 48 centers. 2.8% had major infection (2.6% septicemia, 0.3% mediastinitis, and 0.09% endocarditis). Mortality and postoperative length of stay were greater in those with major infection (mortality, 22.2% versus 3.0%; length of stay >21 days, 69.9% versus 10.7%). Young age, high complexity, previous cardiothoracic operation, preoperative length of stay more than 1 day, preoperative ventilator support, and

presence of a genetic abnormality were associated with major infection after backward selection ($p < 0.001$).(3)

The International Quality Improvement Collaborative for Congenital Heart Surgery in Developing World Countries (IQIC) was officially launched in 2008, the targeted 3 key drivers are safe perioperative practice, infection reduction, and team based practice. In 2010-2012, database from twenty-seven sites had verified data of postoperative mortality and infection by Jenkins and colleagues. Of 15, 049 cases of pediatric congenital heart surgery, unadjusted mortality was 6.3% and any major infection was 7.0%. Standardized mortality ratio (SMR) for the overall International Quality Improvement Collaborative for Congenital Heart Surgery in Developing World Countries were 0.71 (95% confidence interval [CI] 0.62–0.81) in 2011 and 0.76 (95% CI 0.69–0.83) in 2012, compared with 2010 baseline. SMRs among 7 sites participating in all 3 years were 0.85 (95% CI 0.71–1.00) in 2011 and 0.80 (95% CI 0.66–0.96) in 2012; among 14 sites participating in 2011 and 2012, the SMR was 0.80 (95% CI 0.70–0.91) in 2012. Standardized infection ratios were similarly reduced. Congenital heart surgery risk-adjusted mortality and infections were reduced in developing world programs participating in the collaborative quality improvement project and registry. Similar strategies might allow rapid reduction in global health care disparities.(4)

Sen AC and colleagues conducted a retrospective review using pediatric cardiac surgical cases (<18 years of age) performed between 2010 and 2012 at 27 participating sites in 16 developing countries. Of 14, 545 cases, 793 (5.5%) had bacterial sepsis and 306 (2.1%) had surgical site infection. In-hospital mortality was significantly higher among cases with infection than among those without infection (16.7% versus 5.3%; $P<0.001$), as were postoperative ventilation duration (80 versus 14 hours; $P<0.001$) and intensive care unit stay (216 versus 68 hours; $P<0.001$).(5)

The International Quality Improvement Collaborative for Congenital Heart Surgery in Developing World Countries (IQIC) launched since 2008 (4), the authors hypothesized that prevalence of major infections following pediatric cardiac surgery in Siriraj Hospital may be less than our previous report in 2005. The preliminary internal survey in Siriraj hospital was performed in 2018 which surprisingly showed high rate of serious infection post cardiac surgery especially central line associated blood streamed infection (CLABSI). Of 326 children who underwent congenital heart surgery in the center in 2018, 17 patients had CLABSI (5.2%). The CLABSI tended to markedly increase in early 2019 which reported of 9 pediatric patients with CLABSI postoperatively in only first quarter of the year especially related to drug resistance bacterial infection such as Carbapenam resistance enterococci (CRE). Thus, Hospital Infectious control and postoperative unit were discussed regarding this major issue. We decided to adapt CLABSI bundle (6) to use in pediatric postoperative cardiac care unit in purpose to reduce the major infection rate post cardiac surgery in children. The CLABSI bundle campaign has been modified from CDC (6-8) and adapted to prevent drug resistance bacterial infection. The campaign has been including;

- Perform hand hygiene before procedure
- Use maximal sterile barrier precautions (i.e., mask, cap, gown, sterile gloves, and sterile full body drape) prior to insert central line.
- Avoid femoral site for central venous cannulation
- Perform daily audits to assess whether each central line is required
- Scrub the access port or hub with friction immediately prior to each use with an appropriate antiseptic With 70% alcohol at least 15 seconds

Additional maneuver to prevent CRE infection has been plus into this CLABSI bundle for example;

- Bathe patients over 2 months of age before going to the theatre with a 4% chlorhexidine preparation + sterile water 1:1

- For patients over 2 months of age who transferred back from the theatre or immediate surgical intensive care unit with endotracheal tube or central line,

- Blood work and culture from all sites must be obtained.

- Strictly contact precaution would be used until the hemoculture reported no growth

- When patient has been transferred, the initial bedspread is used then changed when the patient has been bathed and all port sites are cleaned.

- Clean all port sites such as 3-way, extension tube, intravenous line with 80% alcohol or change all port sites if the patient is stable

- Bathe patients over 2 months of age with a 4% chlorhexidine preparation + sterile water 1:1, dry the patient off and wipe with 2% chlorhexidine cloth (this should be performed when the patient is stable)

-Clean the equipment and bed environment twice daily

The CLABSI bundle campaign has been initiated in Pediatric cardiac care unit (PCCU) since 1 April 2019. The authors again hypothesized that rate of major infection in children post cardiac surgery especially CLABSI would be reduced following this procedure.

Objectives

1. To compare a prevalence of major infections following pediatric cardiac surgery in Siriraj Hospital prior to in CLABSI bundle (1 APR 2018 – 31 MAR 2019) and following the campaign (1 APR 2019 – 31 MAR 2020)
2. To determine risk factors and impacts of major infections following pediatric cardiac surgery in Siriraj Hospital post CLABSI bundle campaign

Study design

This is a retrospective-comparative, observational study in a single center.

Inclusion criteria

All consecutive pediatric patients (0-18 years) who were admitted for cardiac surgery (open,closed,elective,emergency) at Siriraj Hospital between 1 APR 2018 – 31 MAR 2020 were included.

Exclusion criteria

1. patients with autoimmune diseases such as juvenile rheumatoid arthritis, system lupus erythematosus, cancer
2. Preterm infant (gestational age <37 weeks)
3. Other surgery in same operation such as tracheoplasty, gastrostomy

Outcomes definition:

- Postoperative fever definition: body temperature ≥ 38°C within 7 days after cardiac surgery
- Post operative major infections include;

-Blood stream infection

-Pneumonia

-Urinary tract infection

-Mediastinitis

Outcomes measurement: prevalence of major infection pre and post CLABSI bundle

Methods

After receiving approval from the Siriraj Institutional Review Board, the authors will retrospectively review the medical records of all patients, aged 0 to 18 years old, who underwent cardiac surgery in Siriraj Hospital between April 1, 2018 and March 31, 2020 who had postoperative fever with a body temperature

above 38°C within 7 days after the congenital cardiac operation. Preterm babies, patients with underlying autoimmune disease or patients who underwent other organ operation would be excluded from the study. Demographic data, including age, gender, presence of genetic syndrome, diagnosis of congenital heart disease; and intraoperative data, including type of operation, total operative time (time in operating theatre), cardiopulmonary bypass time (CPB time), and aortic cross clamp time (AoX time) will be obtained. The Aristotle Basic Complexity (ABC) scoring system will be applied to account for complexity. A retrospective analysis of the variables and a statistic analysis will be conducted of the preoperative and intraoperative factors, and the surgical outcomes. Postoperative fever and major infection prevalence will be obtained. The mortality rate, length of stay in the ICU, duration of use of ventilator, and duration of use of intravenously inotropic drugs will be recorded as the impact of outcomes. The patients' identifier would be removed before statistical analysis.

Statistical analysis

Continuous variables are presented as mean +/- SD or median (range) and categorical variables as numbers and percentages. Rate of major infection, CLABSI rate, mortality rate, ventilator usage day, length of stay at pre and post CLABSI bundle period will be compared using student T test and survival analysis. For univariate analysis, the unpaired T test or Mann-Whitney U test will be used to compare continuous variables and Pearson's chi-square test to compare categorical variables. For multivariate analysis, logistic regression analysis will be used to analyze risk of major infection and mortality, adjusting for relevant confounders.

Reference

1. Harrison G Weed M, MD, FACPLarry M Baddour, MD, FIDSA, FAHA. Postoperative fever2019. Available from: https://www.uptodate.com/contents/postoperative-fever?search=post%20operation%20fever&source=search_result&selectedTitle=1~150&usage_type=default&display_rank=1.
2. Vijarnsorn C, Winijkul G, Laohaprasitiporn D, Chungsomprasong P, Chanthong P, Durongpisitkul K, et al. Postoperative fever and major infections after pediatric cardiac surgery. J Med Assoc Thai. 2012;95(6):761-70.
3. Barker GM, O'Brien SM, Welke KF, Jacobs ML, Jacobs JP, Benjamin DK, Jr., et al. Major infection after pediatric cardiac surgery: a risk estimation model. The Annals of thoracic surgery. 2010;89(3):843-50.
4. Jenkins KJ, Castaneda AR, Cherian KM, Couser CA, Dale EK, Gauvreau K, et al. Reducing mortality and infections after congenital heart surgery in the developing world. Pediatrics. 2014;134(5):e1422-30.
5. Sen AC, Morrow DF, Balachandran R, Du X, Gauvreau K, Jagannath BR, et al. Postoperative Infection in Developing World Congenital Heart Surgery Programs: Data From the International Quality Improvement Collaborative. Circ Cardiovasc Qual Outcomes. 2017;10(4).
6. Marschall J, Mermel LA, Fakih M, Hadaway L, Kallen A, O'Grady NP, et al. Strategies to prevent central line-associated bloodstream infections in acute care hospitals: 2014 update. Infection control and hospital epidemiology. 2014;35(7):753-71.
7. Hodge AB, Thornton BA, Gajarski R, Hersey D, Cannon M, Naguib AN, et al. Quality Improvement Project in Congenital Cardiothoracic Surgery Patients: Reducing Surgical Site Infections. Pediatric quality & safety. 2019;4(4):e188.

8. Hussain ASS, Ali SR, Ariff S, Arbab S, Demas S, Zeb J, et al. A protocol for quality improvement programme to reduce central line-associated bloodstream infections in NICU of low and middle income country. BMJ paediatrics open. 2017;1(1):e000008.

CASE RECORD FORM

Birth date (D/M/Y) _ _/_ _/_ _ _ _ Sex ☐ Male ☐ Female	
Date at diagnosis _ _/_ _/_ _ _ _	
Date at admission _ _/_ _/_ _ _ _ Age at admission ___Y ___M ___D	
Body weight _____ kg. Height _____ cm. BMI _____ kg/m2	
Diagnosis: ………………………………………………………………………………………………… Single ventricle ☐ No ☐ Yes	
Syndrome ☐ No ☐ Yes………………………………………………………………………………..	
Other diagnosis ☐ No ☐ Yes………………………………………………………………………………	
Asplenia / polysplenia ☐ No ☐ Yes	
Congestive heart failure ☐ No ☐ Yes	
Pulmonary hypertension ☐ No ☐ Yes	
Previous CVT surgery ☐ No ☐ Yes ………………………………………	
Major infections within 3 months pre-op ☐ No ☐ Yes ………………………………………	
Pre-op status Functional Class …… On ventilator pre-op ☐ No ☐ Yes Retained central line insertion pre-op ☐ No ☐ Yes Pre-op medication ☐ No	

☐ Yes

………………………………………………………………………………

………………………………………………………………………………

Antibiotic preop (beyond prophylaxis) ☐ No

☐ Yes

………………………………………………………

Operation

…………………………………………………………………………………………………..

Aristotle Basic Complexity Score (2006)…………………..

Type onset: ☐emergency ☐urgency ☐elective

Type ☐ closed heart ☐ open heart surgery

Operation time ………………min

CBP ………… min X clamp ………… min Arrest time ……………. min

Peri-op complication………………………………………………

Required ECMO post-op ☐ No ☐ Yes

Required CRRT / PD post-op ☐ No ☐ Yes

Open chest requirement ☐ No ☐ Yes

Total open chest day …………….. days

Requirement of re-operation in the same setting ☐ No ☐ Yes

Post-op antibiotic beyond prophylaxis ☐ No

☐ Yes ……………………………………..

……………………………………..

……………………………………..

……………………………………..

Post-op fever ☐ No

☐ Yes: onset ☐ <1days

☐ 1day-1week

Pattern of fever ☐ Intermittent ☐ Remittent ☐ Persistent

Duration of fever ……………. Days

Note

……………………………………………………………………………………………

……………

……………………………………………………………………………………………

……………

……………………………………………………………………………………………

……………

Post-op infection ☐ No

☐ Yes : ☐ Surgical site infection

☐ Pneumonia

☐ Urinary tract infection

☐ Septicemia

☐ Mediastinitis

☐ Other…………………………………………………

Date operation _ _/_ _/_ _ _ _ …………………………………………………

CBC day 1 Hct …..% WBC …… Platelet ………… ANC …………… PT…………, PTT……………..

CBC Fever day1 Hct …..% WBC …… Platelet ………… ANC …………… PT………, PTT…………

CBC worst Hct …..% WBC …… Platelet ………… ANC …………… PT…………, PTT……………..

Presence of DIC ☐ No ☐ Yes

Presence of hypoglycemia ☐ No ☐ Yes

Presence of septic shock ☐ No ☐ Yes

CRP max ………….. mg/dl date _ _/_ _/_ _ _ _

ESR max ……………. mm/hr date _ _/_ _/_ _ _ _

Procalcitonin max …………. date _ _/_ _/_ _ _ _

Lactate max ……………..... date _ _/_ _/_ _ _ _

BUN max ……………mg/dl date _ _/_ _/_ _ _ _

Cr max ………………mg/dl date _ _/_ _/_ _ _ _

H/C 1 date _ _/_ _/_ _ _ _……………………………………………

H/C 2 date _ _/_ _/_ _ _ _……………………………………………

H/C 3 date _ _/_ _/_ _ _ _……………………………………………

Sputum C/S 1 date _ _/_ _/_ _ _ _……………………………………………

Sputum C/S 2 date _ _/_ _/_ _ _ _……………………………………………

Sputum C/S 3 date _ _/_ _/_ _ _ _……………………………………………

Urine C/S 1 date _ _/_ _/_ _ _ _……………………………………………

Urine C/S 2 date _ _/_ _/_ _ _ _……………………………………………

Urine C/S 3 date _ _/_ _/_ _ _ _……………………………………………

CSF C/S 1 date _ _/_ _/_ _ _ _……………………………………………

CSF C/S 2 date _ _/_ _/_ _ _ _……………………………………………

CSF C/S 3 date _ _/_ _/_ _ _ _……………………………………………

Others culture :

………………. date _ _/_ _/_ _ _ _……………………………………………

………………. date _ _/_ _/_ _ _ _……………………………………………

………………. date _ _/_ _/_ _ _ _……………………………………………

…………………………………………………………………………………………………

…………………………………………………………………………………………………

…………………………………………………………………………………………………

……………………………………………

Outcomes

Date at discharge __ __/__ __/__ __ __ __

Hospital LOS …………………..……… days

Ventilator usage day ………………..….. days

Total central line usage day ……………..days Sum of central line sites

………………

Drug resistance

MDR ☐ No ☐

Yes……………………………………………………………………..

EDR ☐ No ☐

Yes………………………………………………………………………

PDR ☐ No ☐

Yes………………………………………………………………………

MOD ☐ No ☐

Yes…………………………………………………………………………

Neuro-deficit post-op:

- Stroke ☐ No ☐ Yes
- Seizure required AED ☐ No ☐ Yes
- Bed ridden status prior to discharge ☐ No ☐ Yes
- Loss limb (gangrene) ☐ No ☐ Yes

Functional Class prior to discharge ………………………………

In hospital post-op mortality ☐ No

☐ Yes

Date of death __ __/__ __/__ __ __ __
